# More Income, Less Pollution? How Income Expectation Affects Pesticide Application

**DOI:** 10.3390/ijerph19095136

**Published:** 2022-04-23

**Authors:** Xiaoshan Su, Jingyi Shi, Tianxi Wang, Qinghui Shen, Wentao Niu, Zhenzhen Xu

**Affiliations:** 1 School of Management, Zhengzhou University, Zhengzhou 450001, China; suxiaoshan@zzu.edu.cn (X.S.); shi__jingyi@163.com (J.S.); shenqh1022@163.com (Q.S.); 2 Business School, University of Edinburgh, 29 Buccleuch Place, Edinburgh EH8 9JS, UK; s1819714@ed.ac.uk; 3 School of Architecture and Built Environment, Deakin University, Geelong 3219, Australia; xuzhenz@deakin.edu.au

**Keywords:** pesticide reduction, farmer response behavior, value perception, theory of planned behavior

## Abstract

Farmers are still the foundation of China’s current “small, scattered, and weak” agricultural production pattern. As such, increasing guidance for reduction response behavior is central to reducing agricultural pesticide use. Following this pesticide reduction logic, four of the most widely promoted pesticide reduction technologies, including light trapping, biopesticide application, healthy crop growth, and insect-proof net technologies, were selected, and a theoretical analysis framework of farmers’ willingness to adopt these technologies was constructed based on the theories of value perception and planned behavior. An ordered logistic regression model is used to explore key factors behind current pesticide reduction technology perceptions, technology response willingness, and behavioral decisions of farmers in China, with survey data from 516 farmers in Henan Province. The results show that among the four pesticide reduction technologies, healthy crop growth technology is the most-appealing one for farmers, followed by insect-proof net technology and biopesticide application technology. The least-appealing one for farmers is the light trapping technology. Farmers’ perceived degree of income improvement from technology adoption is the main determinant of their willingness, which is positively significant at a 1% confidence level in all four models. In addition, farmers’ willingness to respond to technologies is also significantly influenced by farmers’ perception of technical operational ability, perception of risk from adopting technology, government-related subsidies, government technical training guidance, trust in government promotion of technology, and perception of the government’s role in improving the external environment for adopting technology.

## 1. Introduction

Overreliance on chemical pesticides, together with long-term excessive and inefficient use of pesticides in small-scale decentralized agriculture, has led to a series of problems such as non-point source pollution, environmental damage, declines in agricultural product safety, and risks to human and animal safety [1]. Therefore, more attention should be paid to protecting consumers and field workers (farmers) from the risks to their health and harmful effects of using pesticides [2] and ingesting them with food and drinking water [3]. The Chinese government is concerned with the excessive and inefficient application of pesticides and has introduced a series of policies to guide and reduce pesticide use in agriculture. In February 2021, the “Guiding Opinions of the State Council on Accelerating the Establishment and Improvement of a Green, Low-Carbon and Circular Development Economic System” and the “Central Document No. 1” (the first policy statement released by central authorities each year) were released. Both documents proposed to “accelerate green development of agriculture” and emphasized this paradigm shift as a priority. Since the Ministry of Agriculture issued the “Action Plan for Zero Growth in Pesticide Use by 2020” in 2015, the demand for pesticides in China has shrunk steadily. In 2019, the commodity quantity of pesticides used in China was 1.456 million tons, down 3.2% compared with that in 2018. In 2020, the pesticide utilization rate of China’s three major staple foods reached 40.6% (Ministry of Agriculture and Rural Affairs, 2021), with an increase of 4% over 2015. However, the pesticide application rate in China is not only much higher than the world average but also far exceeds the optimal level in the economic sense [4]. Therefore, there is still ample opportunity to reduce the quantity of formulated product applied as well as the increase application efficiency. Thus, it is necessary to explore the path of pesticide reduction in depth.

In literature, researchers have focused on pesticide reduction from four main perspectives. The first one is the farmer’s perspective, which mainly includes the characteristics of resource endowment and psychological perceptions. Farmers’ decision-making behavior around reducing pesticide use is affected by the gender of the household head, age [5], education level, family planting size [6], degree of part-time employment [7], health status [8], number of family laborers, source of family income [9], identity characteristics [10], etc. In addition, farmers’ expected cost savings and income improvement [11], ecological environment perceptions [12], risk preferences [1], public image [13], learning and operating ability perceptions, and sense of moral responsibility [14] can also significantly affect their decision-making behaviors on reducing pesticides. Among these factors, farmers are more inclined to focus on whether reducing pesticide use can optimize their private economic goals by saving costs and increasing income [11]. Moreover, farmers’ understanding of the relationship between pesticide use and their own health can also influence their decisions to reduce pesticides. Empirical studies have found that farmers’ health awareness is one of the most important determinants influencing the intensity of pesticide application [3,15,16]. Farmers will experience varying degrees of pesticide exposure during pesticide application, which in severe cases can even lead to acute poisoning. About 7% of the agricultural population in the world is affected by pesticide poisoning [17,18]. From 2007 to 2019, only Chongming District, Shanghai, China, reported as many as 1182 cases of pesticide poisoning [19]. The increasing exposure to pesticides has led to an increasing number of farmers perceiving a greater health risk in the process of pesticide application reduction.

The second perspective is based on the organization of the agricultural business. Pesticide reduction is influenced by the characteristics of agricultural business [20]. More moderate-scale agriculture operations [21], more embedded cooperative organizations [22], more service of scale operations, and a better division of labor transactions [23] can effectively contribute to pesticide reduction. The third perspective focuses on the external institutional environment. Agricultural pesticide reduction has a positive economic externality [23], which calls for government intervention to encourage farmers to reduce pesticide use. Government intervention mainly includes policy advocacy, project support [24], government subsidies, a system of proper rewards and punishments [25], institutional trust [26], social capital [27], and social norms [28]. The fourth perspective is the consumer perspective. Consumers’ purchasing preferences can significantly affect agricultural production behavior. Therefore, through green product marketing, health information communication, and other methods [29], consumers can have a stronger passion for green agricultural products, and pesticide use can be reduced [30].

Although pesticide application involves multiple stakeholders, farmers are still the foundation of the “small, scattered, and weak” production pattern of China’s agriculture. Thus, raising farmers’ pesticide reduction awareness is an important logical prerequisite for reducing pesticides in agriculture. Following this logic, many scholars have studied pesticide reduction decision-making behavior from the farmer’s perspective. Wu et al. [31] find that farm size is closely related to farmers’ pesticide use. Their study shows that a 1% increase in farm size induces a 0.5% decrease in pesticide use per hectare. Cheng et al. [21] examine the impact of networks embedded on farmers adopting green farming technologies and find that the role of the family farming area is more substantial than that of education level or annual family income in green technology adoption. Guo et al. [32] study farmers’ pesticide reduction behavior from a mutual perspective of social learning and social network and find that farmers’ production experience and technical knowledge based on their social networks can motivate pesticide reduction behavior. Wang et al. [13] argue that the effect of adopting green agricultural technologies is heterogeneous in terms of risk perceptions and public image, and thus the government should implement various guidance measures accordingly. Zhao et al. [14] study the impact of differences in socio-economic status among farmers on pesticide application behavior based on their sense of moral responsibility and conclude that some social norms such as rules of conduct, regulations, customs, and value standards can encourage farmers to reduce pesticide use.

Xiang et al. [27] find that the accumulation of farmers’ social capital can promote their willingness to adopt fertilizers and technologies for pesticide reduction. Among different dimensions of social capital, social trust plays a major role. In addition, given the obvious lack of incentives for pesticide reduction in China [33], Yang et al. [34] compare the effects of two types of incentives, “green technology training” and “green agricultural subsidies”, on farmers’ biopesticide application behavior. Li et al. [35] argue that promoting green production behavior depend more on value orientation, disciplinary supervision, and internalization of transmission, as well as guiding and incentive regulations. They argue that restraint regulation is prone to the “relative institutional failure” phenomenon, suggesting the necessity to build an interactive regulatory system that integrates formal and informal institutions. Yang et al. [30] argue that agricultural socialization services can effectively boost farmers’ participation in reducing agricultural pesticide use, and Qin et al. [36] argue that market agents such as cooperatives, contractors, and purchasers can also effectively curb excessive pesticide use among farmers.

**Figure 1:**
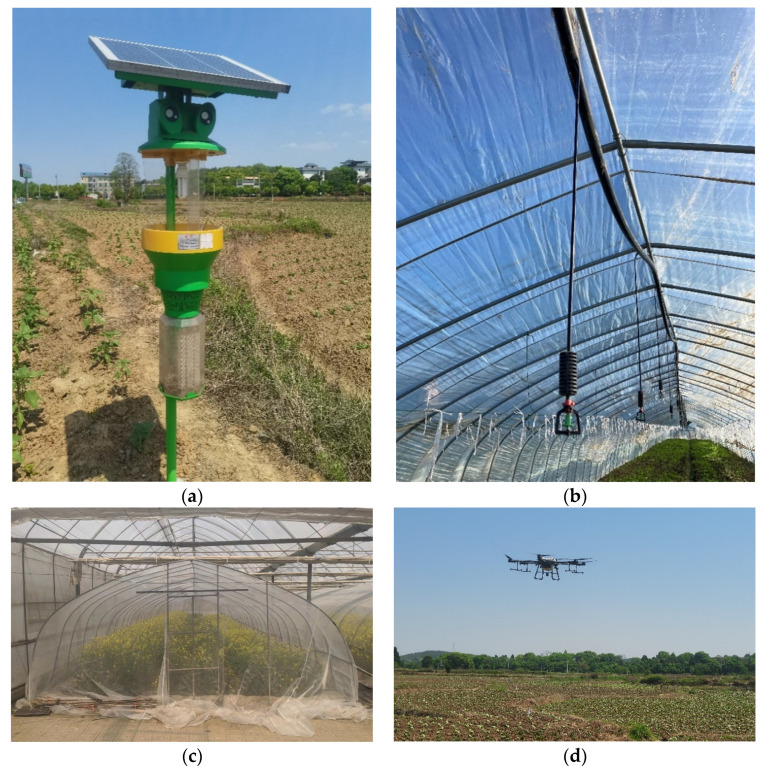
Related images to pesticide reduction technology. (Source: Photo by the author.). Note: (**a**) Light trapping technology (solar wind type insect trap for vegetable fields). (**b**) Healthy crop growth technology based on scientific water and fertilizer management (water and fertilizer sprinkler irrigation). (**c**) Insect-proof net technology (insect-proof net for vegetable greenhouses). (**d**) Drones apply biopesticides in tobacco field (Validamycin + Vivo-Bacillus cereus Mixture).

Compared with traditional high-toxicity and high-residue chemical pesticide application technology, pesticide reduction technology is a technology to reduce and control pests with higher efficiency. It uses physical, biological, ecological, and other pest prevention and control methods to replace traditional chemical pesticides. It aims to reduce the amount of pesticide application and improve the efficiency of pest control [37]. It is characterized by low toxicity, low residue, high efficiency, and low dosage requirements. Common physical prevention and control technology mainly includes light trapping technology and insect-proof net technology, which takes advantage of the characteristics of insects (e.g., phototaxis) to trap and kill crop pests. Biological prevention and control technology mainly includes biopesticide application technology and natural enemy preying, which controls insects and bacteria by using insects and bacteria themselves. Ecological prevention and control technology mainly includes improving water and fertilizer management and promoting farmland ecological engineering, intercropping and other biodiversity control methods of healthy crop growth technology, and artificially enhancing crop resistance to pests and diseases (as shown in Figure 1). Pesticide reduction technologies not only help reduce the health risks to farmers but also help improve the ecological environment and ensure the safety of agricultural products, and promote high-quality agricultural development [38].

The above studies on farmers’ pesticide reduction behavior provide a rich theoretical foundation and empirical basis for this paper. In this study, the four most widely used pesticide reduction technologies, including light trapping, biopesticide application, healthy crop growth, and insect-proof net technologies, are selected, and a theoretical framework of farmers’ willingness to adopt pesticide reduction technologies is constructed based on the theories of value perception and planned behavior. This study also takes into account the current status of the adoption of pesticide reduction technologies by farmers in China.

This paper uses survey data from 516 farmers in Henan Province to compare and analyze the commonalities and differences in farmers’ willingness and behavior in response to the four existing pesticide reduction technologies. It also reveals the key factors behind farmers’ perceptions of pesticide reduction technology, their willingness to adopt the technologies, and farmer behavioral decisions in China. This paper contributes to a deeper understanding of farmers’ willingness to respond to pesticide reduction technologies in a psychological sense. It also provides empirical implications for the government to promote pesticide reduction and also to maintain efficient production in agriculture.

## 2. Theoretical Analysis Based on the Theories of Value Perception and Planned Behavior

Research on perceived value dates back to Porter, who argues that perceived value is the difference between a decision-maker’s perceived benefits and perceived costs [39]. With this initial claim, scholars define perceived value from different perspectives: the overall utility based on comparing gains and losses [40], the ratio of perceived benefits to perceived costs [41], and the trade-off between benefits and costs in the whole process of multiple transaction behaviors [42]. Nowadays, the view of ‘balance between perceived benefits and efforts’ is widely accepted [43]. Since then, some researchers have started to study farmers’ production behavior based on a value perception perspective. To explore the differences and influencing factors of farmers’ behaviors and willingness, they analyze the costs and benefits behind farmers’ behavioral decisions mainly based on perceived benefits and perceived risks [44,45], or construct farmers’ value systems from different value dimensions such as economic dimension, ecological dimension, emotional dimension [46,47], monetary dimension, social dimension, conditional dimension, and perceived dimension [48].

Numerous studies have shown that farmers’ value perceptions significantly affect their willingness to adopt technologies [49,50], and generally, the stronger the value perception, the stronger the willingness [51]. Among them, expected benefits and integrated value perceptions have positive effects [52], while cost and benefit risk perceptions have a negative effect [53].

The theory of planned behavior, proposed by Ajzen [54], suggests that behavioral attitudes, subjective norms, and perceived behavioral control can jointly affect an individual’s intentions, which then affect their behavioral outcomes. Behavioral attitudes arise from the people’s expected outcome. Subjective norms are pressures and constraints from other individuals or organizational groups that make people perform or not perform a behavior. Subjective norms include ‘legal norms’ and ‘descriptive norms’ [55]. Perceived behavioral control is an individual’s prior self-perception of the ease or difficulty of performing a behavior based on past experience and future expectations. It is influenced by the individual’s perception of their own resources, skills, opportunities, and other factors [56].

In general, the more positive the behavioral attitude of the subject is, the greater the subjective normative constraints are, and the stronger the perception of behavioral control and the intention to perform a certain behavior is. The theory of planned behavior has been widely used in research on farmers’ production behavior decisions because of its explanatory power in human’s general decision-making behavior [54].

Based on the theory of planned behavior, Xie et al. [57] explore the intrinsic attribution of the heterogeneity in farmers’ willingness to adopt ecological farming, and Hu et al. [58] studied farmers’ heterogeneous willingness to adopt rice and shrimp co-cropping models. Some researchers further expand the research framework of this theory, however, because they argue that the theory of planned behavior does not explain the deviation between the empirical results and the actual behavior of farmers [59]. In addition to behavioral attitudes, subjective norms, and perceived behavioral control factors, Shi et al. [60] introduce economic rationale and environmental values to study the factors influencing farmers’ willingness to adopt green production. Shi and Yu [61] introduce risk expectations and perceptions of citizenship to study the mechanisms of farmers’ homestead withdrawal and analyze the moderating factors behind farmers’ behavioral decisions. Zhang et al. [49] analyze the factors influencing farmers’ adoption of straw return technology and its relation with external variables such as perceived value and awareness of environmental responsibility based on an extended Technology Acceptance Model (TAM). In integrating the theories of value perception and planned behavior, this paper constructs a theoretical, analytical framework for farmers’ willingness to adopt pesticide reduction technologies based on three key factors: behavioral attitudes, subjective norms, and perceived behavioral control, as described below.

Farmers’ behavioral attitudes, subjective norms, and perceived behavioral control jointly influence their willingness to adopt pesticide reduction technologies. Behavioral attitudes are farmers’ expectations and evaluations of the outputs of adopting pesticide reduction technology, which is formed based on farmers’ value perception of technology adoption, risk perception, and perception of technical operational ability, and may also be related to farmers’ personal characteristics [28]. Three aspects of farmers’ value perception of technology adoption are reflected in their personal characteristics: economic values, ecological values, and social values.

**Figure 2:**
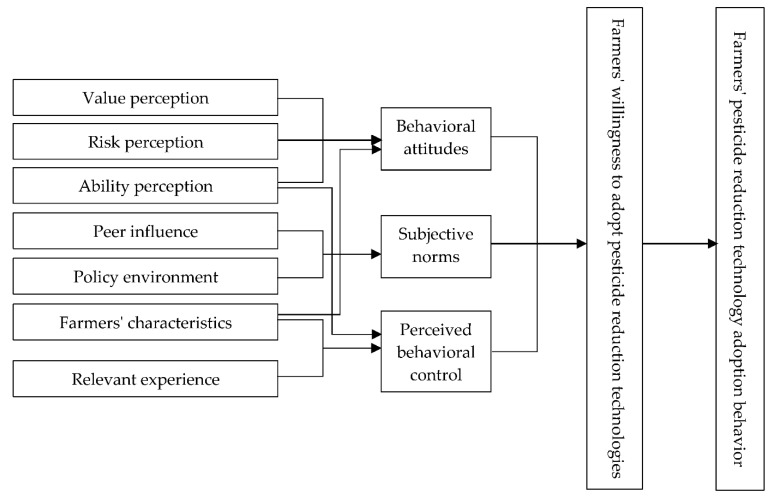
Theoretical framework of farmers’ response behavior to adopting pesticide reduction technology.

Subjective norms are a collection of various external constraints farmers face when deciding to adopt pesticide reduction technology. The effect of subjective norms can be understood as the interactive influence of people around and the policy environment in which farmers live. Policy environment factors include government subsidies, publicly available government technology information, government-related technical training guidance, and the role government plays in improving the external environment for adopting technology. Perceived behavioral control is a farmer’s psychological perception of the difficulties in the practical application of pesticide reduction technologies. This psychological perception is also related to the farmer’s personal characteristics, production conditions, relevant experience, and perception of technical operational ability. The interactive relations among all these factors are shown in Figure 2.

## 3. Data Sources and Model Construction for Farmers’ Responses to Pesticide Reduction Technologies

### 3.1. Data Sources

**Figure 3:**
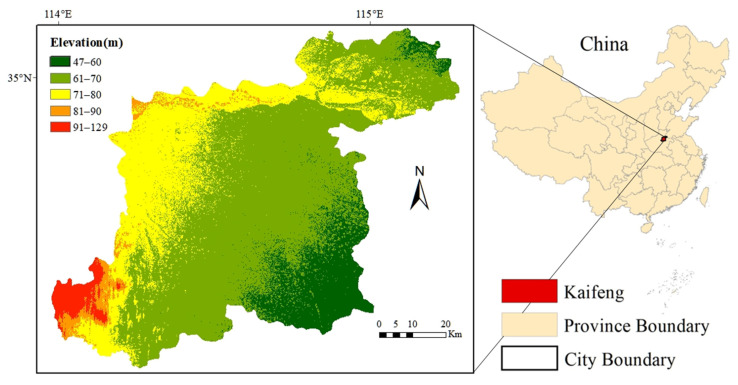
Topographic location of Kaifeng, Henan Province, China.

The data for this study are mainly collected from a field survey of farmers conducted by the research team in Kaifeng, Henan Province (as shown in Figure 3) from July to September 2020, with questionnaires (as shown in Supplementary Materials) and interviews by the trained researchers. Six villages in the administrative area of Kaifeng were randomly selected for field research. The survey focuses on farmers’ willingness to adopt pesticide reduction and pest control technologies in their agricultural production process. The contents of the survey mainly include farmers’ basic characteristics, production conditions, perceptions and psychology around pesticide reduction technology, relevant experience of technology adoption, the current status of technology response, and policy perceptions. A total of 516 valid observations were obtained from this survey.

### 3.2. Basic Characteristics of Samples

Table 1 shows that most of the farmers in the sample are middle-aged and elderly people with a relatively low level of education. The proportions of male and female farmers interviewed are similar, accounting for 54.8% and 45.2% of the total sample, respectively. The farmers’ age varies from 20 to 78 years old, and those who are above 45 years old account for 73.4% of the total sample. The education level of farmers is generally low; 80.6% of them only have a primary school (43.6%) and junior/middle school education. Moreover, most farmers have been engaged in agricultural production for a long time, and 76.7% of farmers have more than 25 years of experience in agricultural production.

The overall family planting area of the sample farmers is of small or medium size. A total of 62.6% of farmers have a planting area that is between 6 and 15 mu (1 mu = 0.067 hectares). The planting area for 17.6% of the farmers is less than 5 mu. Farmers whose planting area is more than 35 mu account for only 3.9%. In total, agricultural income is the major source of family income for 76.7% of the sample farmers. A total of 36.2% of farmers claim that pesticide expenditure is a substantial component of total family agricultural expenditure. It is not common for the sample farmers to have either part-time employment or membership in cooperatives. More specifically, 36.2% of farmers have part-time employment, and 42.1% of farmers are members of professional cooperatives. A total of 97.1% of the sample farmers have ever encountered technical problems in the process of agricultural production. The above descriptive statistics are in line with the current situation of agricultural production in underdeveloped regions of China.

### 3.3. Model Construction

The question on willingness to adopt technology in the questionnaire has three options: “unwilling”, “doesn’t matter”, and “willing”. This feature makes the answer an ordered multi-classification variable. Therefore, this study chooses an ordered logistic regression model for quantitative analysis. The model formulations are as follows:

(1) $$In(\frac{p(y\leq j|x)}{1-p(y\leq j|x)})=\mu_{j}-(\alpha +\sum_{i=1}^{k}\beta_{i}x_{i})$$

(2) $$p(y\leq j|x)=\frac{e^{\mu j-(\alpha +\sum_{i=1}^{k}\beta_{i}x_{i})}}{1+e^{{}^{\mu j-(\alpha +\sum_{i=1}^{k}\beta_{i}x_{i})}}}\;$$

where y denotes the dependent variable, representing farmers’ willingness to adopt pesticide reduction technologies; $x_{i}$ denotes the explanatory variable, representing the ith factor influencing farmers’ adoption of pesticide reduction technologies; μ denotes the threshold or critical value; α denotes the intercept; and $\beta_{i}$ denotes the corresponding parameter to be estimated for $x_{i}$, representing the degree and direction of the influence of each explanatory variable on the dependent variable.

### 3.4. Variable Selection

#### 3.4.1. Dependent Variables

The pesticide reduction technologies involved in this study mainly focus on four types of pesticide reduction: light trapping technology, biopesticide application technology, healthy crop growth technology, and insect-proof net technology. Four ordered logistic regression models are constructed to study the factors affecting farmers’ willingness to adopt pesticide reduction technologies. The dependent variable is farmers’ willingness to adopt each technology. There are three options for measuring farmers’ willingness: “unwilling”, “doesn’t matter”, and “willing”, which correspond to values 1, 2, and 3, respectively.

#### 3.4.2. Explanatory Variables

“Farmers” are the interested subjects that are expected to respond to the promotion of pesticide reduction technologies in this study. Indicator designs related to farmers have been widely used and proven effective. Li et al. [35], Guo et al. [62], and Gao et al. [63] describe the individual characteristics of farmers in terms of gender, age, education level, years of agricultural production, part-time employment and farmer status; Tian et al. [6], Li et al. [22], and Li et al. [64] describe the family characteristics of farmers in terms of family planting size, income structure, and cost expenditure. Huang et al. [25], Yan et al. [38], and Zhang et al. [65] describe farmers’ profit perceptions in terms of income improvement, ecological environment improvement, and agricultural product safety; Huang et al. [11], Wang et al. [13], and Su et al. [66] describe farmers’ risk perceptions in terms of operational risk, market risk, and perception of operational ability. Cheng et al. [21], Gai et al. [24], Xiang et al. [27], He et al. [67], and He et al. [68] describe the policy environment in terms of government subsidies, technical information publicity, technology training, interpersonal trust, and government credibility. Based on previous literature, this study selects 21 indicators on farmer characteristics, production conditions, perceived value and risk of technology adoption, ability perception, peer influence, and policy environment, as well as relevant experience to construct a model including the factors influencing farmers’ response to pesticide reduction technologies. These indicators can be classified into eight categories. A conceptual description of these indicators is found in Table 2.

First, the farmer characteristics category mainly includes two factors: personal features and production conditions. Among them, four indicators are selected for personal features: the gender of the household head, age, education level, and years of production experience. Three indicators are selected for factors of production conditions: the size of the family planting land, the income structure, and the proportion of pesticide expenditure in total family agricultural expenditure. Age, gender, and education level of the household head are expected to affect farmers’ technology response behavior, and the expansion of family planting land is expected to increase the risk of technology adoption by farmers. Farmers with larger agricultural income tend to be more cautious in technology adoption, and a higher proportion of pesticide expenditure in total family agricultural expenditure predicts a higher likelihood of farmers adopting pesticide reduction technologies.

Second, farmers’ psychological perception consists of three categories of technology adoption: value perception, risk perception, and ability perception. Five indicators related to technology adoption are selected: farmers’ perception of the degree of income improvement, perception of improved ecological environment, perception of product safety, farmers’ attitudes towards risk, and perception of technical operational ability. Pesticide reduction technologies have substantial value on ecological, economic, and social, and farmers’ motivation to pursue profit and food and ecological safety will promote their technology response behavior. Meanwhile, pesticide reduction technologies are also characterized by uncertain effects, complex technical operations, and high market operation risks. This cognitive conflict brings a significant impact on farmers’ technology response behavior: stronger risk perception generally leads to lower technology adoption [13].

Third, subjective norms include peer influence and relevant policy environment factors. The policy environment is measured by five indicators: farmer satisfaction with government subsidies, satisfaction with technical information publicized by the government, satisfaction with government technical training guidance, trust in government promotion of technology, and perception of the government’s role in improving the external environment for technology adoption.

Fourth, the category of relevant experience includes three aspects that can affect farmers’ technology response behavior: cooperative organizations, part-time employment, and relevant experience of farmers. Therefore, three indicators, participation in professional cooperatives, part-time employment, and the frequency of technical problems encountered in industrial operations, are selected as other variables.

## 4. Analysis of Farmers’ Response Behavior to Pesticide Reduction Technologies

### 4.1. Farmers’ perceptions of Pesticide Reduction Technologies

Based on the statistical analysis of the sample farmers’ psychological perceptions of pesticide reduction technologies (Table 3), the farmers generally agree that the four pesticide reduction technologies have a higher safety level in terms of ecology and product quality. Among them, farmers strongly emphasize the ecological safety of the healthy crop growth technology, with 84.9% of farmers believing it is relatively safe, followed by light trapping technology. However, 11.2% of farmers are skeptical about the biopesticide application technology, considering it is relatively unsafe ecologically. However, based on the benign nature of new biopesticides, such should be the most desirable green option at present [69], which indicates that the relevant government departments need to further strengthen technical publicity and continuously improve farmers’ perceptions of this technology.

The vast majority of farmers believe that adopting pesticide reduction technologies have helped to increase agricultural income. The scale of the impact, however, is considered to be relatively negligible. The vast majority of farmers considered that technology adoption has a “relatively large” or “very large” impact on income growth. A total of 26.5% of the sample farmers think the impact of healthy crop growth technology is substantial, followed by the biopesticide application technology (10.9%). However, only a few of them believed that adopting the biopesticide application technology would have a “very large” impact on agricultural income growth.

Due to the wide variety of biopesticides’ poor stability and complex application process [70], it is necessary to scientifically select proper biopesticide products and suitable equipment [71]. At the same time, it is also essential to accurately grasp the time, dose, and time interval of application; otherwise, it will be less effective and even induce additional cost [72]. Therefore, farmers generally consider that biopesticide application technologies are difficult to operate [73] and require training on the application technologies of different biopesticide varieties. In the sample of this paper, similar observations were obtained. The biopesticide application technology is said to be the most difficult one, with 40.3% and 26.6% of farmers saying it is “relatively difficult” and “very difficult”, respectively. The healthy crop growth technology is considered to be the easiest one to implement. The survey finds that farmers are risk-sensitive, and the majority of farmers believe that there are risks in adopting pesticide reduction technologies. Among them, most farmers believe that the risks of the biopesticide application technology are “relatively large” or “very large”, accounting for 51.4% and 6.6% of the sample, respectively. By contrast, the adoption of insect-proof net technology is considered to be the least risky among the four pesticide reduction technologies.

### 4.2. Farmers’ Willingness and Behavior in Response to Pesticide Reduction Technologies

In order to clarify farmers’ attitudes to pesticide reduction technologies, this study further surveys and interviews the sample farmers with additional questions focusing on four aspects: “whether they have heard”, “whether they are concerned”, “ whether they need”, and “whether they are willing” (Table 4). The results show that:

(1) 65.5% of farmers have heard of these four pesticide reduction technologies. The most well-known ones are insect-proof net technology and biopesticide application technology, which reflects the efforts to promote them by relevant government departments.

(2) Farmers show the greatest interest in the healthy crop growth technology (e.g., soil test and formula fertilization, crop rotation and intercropping, deep loosening and tilling of the soil, etc.). The proportion of concern and need reached 80.6% and 74.2%, respectively, followed by insect-proof net technology.

(3) Light trapping technology is the least attractive option for farmers, and the percentage of those unwilling to adopt the technology is also the highest (79.8%). One possible reason for this is that they are skeptical about the insecticidal effect. Moreover, the insecticidal equipment needs to be set up in the field, which can induce many accompanied problems in practice.

(4) Among the respondents, the number of farmers who are willing to adopt the insect-proof net technology is the largest, accounting for 73.6% of the total sample. By contrast, the number of farmers who are unwilling to adopt it is also relatively substantial (19.8%). Although the insect-proof net technology is relatively easy to apply, farmers also express greater difficulties in selecting suitable specifications for insect-proof nets, choosing the follow-up of supporting measures for covering and cultivation of insect-proof nets, and selecting suitable varieties of agricultural products.

(5) In total, 58.3% of the interviewed farmers claim they are reluctant to adopt the biopesticide application technology, which is the second-highest proportion among the four technologies after light trapping. The reasons for this might be the high cost of biopesticide application, its poor quick-acting properties, narrow insecticidal and bactericidal spectrum [12], and relatively complex application procedures. These factors make farmers think that the technology is less cost-effective and reduce their willingness to adopt it.

## 5. Factors Influencing Farmers’ Responses to Pesticide Reduction Technologies

### 5.1. Correlation Analysis of Independent Variables and Willingness to Utilize Pesticide Reduction Technologies

SPSS20.0 is used to analyze correlations in the survey data, and Kendall’s Tau-b coefficients of the independent variables and farmers’ willingness to respond to light trapping technology, biopesticide application technology, healthy crop growth technology, and insect-proof net technology are obtained separately. For brevity, only the results of the analysis related to the willingness to respond to the light trapping technology are presented in Table 5.

The results show that farmers’ age, participation in professional cooperatives, years of working in agricultural production, perception of income improvement, peer influence, satisfaction with government-related subsidies, satisfaction with technical information publicized by the government, trust in government promotion of technology, satisfaction with government technical training, and perception of the government’s role in improving the external environment for technology adoption are all significantly and positively correlated with farmers’ willingness to implement pesticide reduction and pest control technologies. In contrast, gender, part-time employment, perception of operational ability, and risk perception are negatively correlated with farmer willingness. The results may indicate that farmers can be prompted to respond to pesticide reduction technologies by improving the government-related subsidy system, enhancing government-related technical information publicity, and training guidance. These measures can lower the risk of technology adoption and optimize the external environment for technology adoption.

### 5.2. Analysis of Influencing Factors of Farmers’ Responses to Pesticide Reduction Technologies

The ordered logistic regression models of farmers’ responses to the four technologies are estimated using the stepwise regression analysis method in Stata 12.0. The results are shown in Table 6, Table 7, Table 8 and Table 9. Combined with the goodness-of-fit test index of each model, the model chi-square statistic is significant at the 1% level, which indicates that the models are powerful in predicting the dependent variable and the effects of the explanatory variables are strong.

First, the influence of farmer characteristics on their willingness to respond to pesticide reduction technologies is discussed in terms of the following aspects. Among the results of the four ordered logistic regression models, only age and farmers’ years of working in agricultural production significantly affect farmers’ willingness to respond to the insect-proof net technology. The effect is positively significant at a 1% confidence level. This result indicates that older farmers who have been in agricultural production longer are more willing to respond to the insect-proof net technology. The government has been promoting the insect-proof net technology for several years, and the technology is relatively easy to apply, so older farmers who are more experienced in agricultural production have better knowledge and richer experience with it. Therefore, they can better solve the problems of suitable insect-proof nets selection, soil disinfection, wind, and flood prevention, etc., which can more effectively improve the effectiveness of insect-proof nets.

Second, the impact of farmers’ psychological perception on their willingness to respond to pesticide reduction technologies is as follows. Farmers’ perception of the degree of income improvement from technology adoption is positively significant at a 1% confidence level in all four regression models, indicating that the more the technology response contributes to agricultural income improvement, the more willing farmers are to adopt the technology. Farmers’ perception of technical operational ability significantly influenced their willingness to respond to healthy crop growth and insect-proof net technologies, and the effect is negatively significant for both technologies at the 1% confident level. This indicates that the harder the technology is perceived to operate, the less willing farmers are to adopt the technology. Farmers’ perception of technology adoption risk significantly affects their willingness to respond to light trapping and insect-proof net technologies. The effect is significant at a 1% level for both technologies. The biopesticide application passes the significance test at the 5% level, both in a negative direction, indicating that the greater the perceived risk of technology adoption is, the less willing farmers are to respond to the technologies.

Agricultural production has many uncertainties. Farmers adopting new agricultural technologies are exposed to not only natural and social risks but also market and technological risks. Therefore, reducing risks and maximizing returns is the fundamental motivation for farmers’ behavioral decisions. In promoting pesticide reduction and pest control technologies, on the one hand, it is necessary to vigorously advertise the efficient, environment-friendly, safe, and harmless features of these technologies to enhance farmers’ confidence in technology adoption through word-of-mouth publicity through demonstration households and also enhance their perception of technical operational ability through field demonstrations. On the other hand, the adoption of some technologies will improve product quality by sacrificing yield to some extent. This trade-off requires the government to optimize planting structures, which refer to the combination and optimization of different varieties of crops guided by the local government through better regulations. It also requires the government to actively seek markets for products to ensure farmers’ income. In this way, farmers will be more willing to respond to pesticide reduction technologies.

Third, the influence of subjective norms on farmers’ willingness to respond to pesticide reduction technologies also deserves researchers’ attention. Among the environmental policy factors, farmers’ satisfaction with government subsidies is positively significant at a 1% confidence level in all four regression models. Farmers’ satisfaction with government technical training guidance significantly affects their willingness to respond to light trapping, biopesticide application, and healthy crop growth technologies, with a positive significance level of 1%. The estimated effect of farmers’ trust in government promotion of technology is positively significant at the 1% level in the response model for biopesticide application and insect-proof net technologies and is positively significant at the 5% level in the response model for light trapping and healthy crop growth technologies. Farmers’ perception of the government’s role in improving the environment for technology adoption is positively significant at the 1% level in the biopesticide application technology response model.

These results show that a higher level of farmers’ satisfaction with the government-related subsidy system and the effectiveness of government technical training, a higher level of farmers’ trust towards government-promoted technologies, and a more crucial role of the government in improving the external environment for technology adoption, can make farmers more willing to respond to new technologies. As “rational economic individuals”, farmers make their decisions by comparing costs and benefits. In general, the lower the cost, the higher the expected benefits. After technology adoption, they can attain more compensation from the government to hedge the risk of technology adoption. The government can improve the environment for technology adoption with more detailed and clearer technical training guidance and improve the corresponding infrastructure. Meanwhile, farmers will be more convinced that the technologies can improve productivity if the government is more credible. Consequently, they will have stronger motivation to adopt pesticide reduction technologies.

Fourth, the impact of relevant experience on farmers’ willingness to respond to pesticide reduction technologies is as follows. The frequency of the technical problems encountered is negatively significant at the 5% level in the model of willingness to respond to light trapping technology, indicating that frequent technical problems in practice hinder farmers’ willingness to respond to light trapping technology. Although the light trapping technology has been used for decades, the function of insecticidal lamps has not seen substantial improvements for many years [74]. Moreover, many technical problems arise in farmers’ long-term practice [75], which then affects farmers’ willingness to respond to the technology.

## 6. Conclusions

### 6.1. Main Conclusions

Based on the survey of 516 farmers in Henan Province, this paper constructs four ordered logistic regression models for light trapping, biopesticide application, healthy crop growth, and insect-proof net technologies to explore farmers’ willingness and determinants of their response to pesticide reduction technologies. Three main conclusions are drawn below.

First, the interviewed farmers have a high perception of the ecological safety and product safety of all four pesticide reduction technologies, and most farmers are familiar with the four technologies to some degree. The four technologies can be ranked from high to low in terms of farmers’ concern and need: healthy crop growth technology, insect-proof net technology, biopesticide application technology, and light trapping technology. Farmers are overall passive in adopting new technologies, and most farmers believe the costs are high while the benefits are uncertain. Large-scale demonstrations which can help farmers learn more about the technologies are necessary to increase farmers’ willingness to adopt them.

Second, most farmers believe that adopting pesticide reduction technologies has slightly increased agricultural income, but the impact is relatively small. Among them, 16.9% and 6.6% of farmers believe adopting healthy crop growth technology has a “relatively large” and “very large” impact on income growth. Very few farmers believe adopting biopesticide application technology will have a “very large” impact on agricultural income growth. Moreover, farmers generally believe that biopesticide application technology is the riskiest and hardest technology to apply. The least-appealing technology farmers are unwilling to adopt is the light trapping technology, followed by the biopesticide application technology.

Third, the effects of the farmers’ characteristics, their technology perceptions and psychology, the relevant policy environment, and the relevant experience on farmers’ response to different technologies are heterogeneous. Overall, farmers’ willingness to adopt technologies is mainly affected by their perception of many factors, including income improvement, their technical operational ability, their estimated risks, government-related subsidies, government technical training guidance, trust in government promotion of the technology, and the government’s role in improving the external environment for technology adoption. Most of these factors have positive effects on farmers’ willingness to adopt technologies, while higher risks can inhibit their willingness.

### 6.2. Future Implications and Recommendations

Typically, farmers make decisions in response to technologies by considering whether the technology can reduce productive inputs and labor intensity, save working time, and increase productivity and income. However, with the increasing demand for sustainable environmental practice and product quality, farmers’ technological needs are no longer limited to simply increasing productivity and income. They also begin to emphasize the quality of the products and the sustainability of the ecological environment.

Farmers have an intrinsic motivation to adopt new technologies only when the perceived value of their adoption exceeds the risk of loss. Therefore, encouraging farmers to positively respond to pesticide reduction technologies can start from two key points: first, strengthen technology publicity and demonstrations to guide farmers to form a more positive value perception of technology adoption, thus stimulating their intrinsic motivation to adopt pesticide reduction technologies; second, use technical subsidies, and incentive mechanisms to encourage farmers to more positively respond to pesticide reduction technologies.

First, expand the breadth and depth of technical publicity and training programs to raise farmers’ awareness of reducing pesticide use. Farmers’ perceptions of pesticide reduction technologies, especially whether technology is promising in improving productivity and quality and whether it contributes to high product prices and best-selling products are the key concerns for farmers’ behavioral decisions. Information publicity and training guidance on pesticide reduction technologies are important ways of improving farmers’ perception of pesticide reduction technologies and promoting their technology response behavior [76].

On the one hand, it is necessary to advertise pesticide reduction technologies through multiple channels such as radio, television, the Internet, MMS/texting, technical training courses, etc. These measures can facilitate the top-down publicity and promote the role of government agricultural technology extension departments. They can also highlight the benefits of adopting pesticide reduction technologies, making farmers fundamentally aware of the importance of pesticide reduction technologies to ecological protection and their own health. The prevalence of such information can enhance their confidence in technology adoption [77]. At the same time, the ecological education of farmers should be continuously strengthened to guide their choice of pesticide reduction behavior from the dimension of ecological protection and personal health. On the other hand, through government regulation, efforts should be made to optimize planting structures and actively seek broader markets for products. The incentive mechanisms such as government subsidies should also be improved to ensure farmers’ income, strengthen the direct perception of the results and effectiveness of pesticide reduction technology responses, enhance willingness to respond to technology, and finally promote technology adoption behavior.

Second, promote the innovation of pesticide reduction technologies to balance technology supply and demand and achieve cost savings and income enhancement. A scientific and technological innovation mechanism targeting the needs of farmers should be established and continuously improved. It should highlight the transformation from researchers’ technical innovation to economic outputs and also take into account the public and social nature of pesticide reduction technologies, and facilitate government investment in technological innovation research and development. In this way, the mechanism should be able to contribute to the diffusion and adoption of pesticide reduction technologies through scientific and technological innovations [78]. The technical needs of farmers should be carefully clarified, and the help and guidance provided to the farmer trainees should precisely match their demands and mitigate their difficulties.

The channels of education and training should also be further broadened, and the role of private training institutions, professional associations, and leading enterprises should be used to promote high-quality training programs. At the same time, training contents and methods should be further improved to enhance farmers’ technical mastery and application abilities. After training, additional efforts must be made to improve land cultivation, finance, insurance, and other related supporting systems to promote the adoption of new pesticide reduction technologies.

Third, effectively improve farmers’ satisfaction with technical training and promote farmers’ active participation in technical training. Due to the outflow of young and strong laborers from rural areas to urban areas, most of the individuals actually engaged in agricultural production are older and limited in relevant knowledge. Therefore, more on-site field guidance and instruction should be developed. By making better use of agricultural leisure time and appropriately extending the training time, the government can hire agricultural experts from inside and outside the province to carry out field guidance to improve the applicability and practicality of the technologies and help farmers to operate them proficiently [79]. Farmers who have received such training should also be publicized more vigorously to enhance their demonstration effect and to stimulate other farmers’ intrinsic motivation to participate in agricultural green education and pesticide reduction production skills training.

Fourth, improve the government subsidy system, strengthen institutional trust, and build a favorable environment for technology adoption. Government subsidies play an important role in promoting the adoption of pesticide reduction technologies as an explicit incentive. Farmers’ trust in the government also significantly influences their willingness to adopt pesticide reduction technologies. Further improving the government subsidy system, strengthening government trust-building, and reducing transaction costs and institutional costs in technology promotion can effectively incentivize farmers to adopt pesticide reduction technologies. The importance of pesticide reduction technology subsidies should be highlighted as equivalent to that of other mainstream subsidies such as “direct subsidies for grain cultivation”, “subsidies for good seeds”, “subsidies for the purchase of agricultural machinery”, and “comprehensive subsidies for agricultural materials” so that subsidies can become a regular form of incentive for farmers to adopt pesticide reduction technologies [76].

Relevant policies, laws, and regulations should be strictly implemented to support and benefit farmers. This can help to ensure that compensation or subsidies for pesticide reduction technologies are in place and maintain the credibility of the government. As long as farmers are convinced that they will benefit from adopting the technologies and the compensation or subsidies can hedge the additional costs and risks stemming from using the technologies, they will be more willing to try these technologies [80]. The risk compensation system should be designed to suit those high-income, well-educated, and large-scale planting farmers and also coordinate with differentiated subsidy policies. The transparency of policy implementation must be ensured to establish a foundation of mutual trust and communication between the government and farmers. Such transparency can also mitigate farmers’ worries when participating in environmental protection and governance. The whole system should also focus on solving the problems and obstacles encountered by farmers in adopting pesticide reduction technologies.

Fifth, it is vital to strengthen the training of new professional farmers and solidify the demonstration effect. Stakeholders, including family farms and large professional households of new professional farmers with moderate-scale operations, as well as intensive and specialized production organization structure, provide the favorable potential for applying pesticide reduction technologies. Strengthening the guidance and promotion of pesticide reduction technologies for new professional farmers can become a “field classroom” for other small and scattered farmers, allowing many small farmers to learn operational skills of new pesticide reduction technologies in the field. At the same time, risk-averse small farmers will become increasingly aware of the economic and social benefits of pesticide reduction technologies, which promotes their adoption of pesticide reduction technologies.

In order to strengthen the training of new professional farmers and guide the intensive and specialized operation in agriculture, it is first necessary to clarify farmers’ contracting rights on land, establish a long-term mechanism for land transfer, secure farmers’ land tenure, and create an environment for moderate scale operations; second, a strict approval system should be established, and organizations that are qualified for approval should be allocated differentiated financial support and subsidies according to their operations so that these large demonstration households can truly benefit from the whole process and better exert their demonstration effect [14,81].

## Figures and Tables

**Table 1:** Basic characteristics of sample farmers.

Features	Classification	Frequency	Percentage %
Gender	Male = 1	283	54.8
	Female = 0	233	45.2
Age	≤25 = 1	15	2.9
	26~35 = 2	41	7.9
	36~45 = 3	81	15.7
	46~55 = 4	219	42.4
	≥56 = 5	160	31.0
Education level	Primary school = 1	225	43.6
	Junior/middle school = 2	191	37.0
	Technical secondary school and high school = 3	84	16.3
	College and above = 4	16	3.1
Part-time employment	Working part-time = 1	187	36.2
	Not working part-time = 0	329	63.8
Years of agricultural production	≤5 = 1	10	1.9
	6~10 = 2	40	7.8
	11~25 = 3	70	13.6
	26~39 = 4	289	56.0
	≥40 = 5	107	20.7
Technology problems	Encountered = 1	501	97.1
	Not encountered = 0	15	2.9
Planting size (mu)	≤5 = 1	91	17.6
	6~15 = 2	323	62.6
	16~25 = 3	45	8.7
	26~35 = 4	37	7.2
	≥36 = 5	20	3.9
Income structure	Agricultural income dominated = 1;	396	76.7
	Non-farm income dominated = 0	119	23.1
Professional cooperatives	Joined = 1	217	42.1
	Did not join = 0	299	57.9
Proportion of pesticide Expenditure in total Family agricultural expenditure	Very small = 1	24	4.6
	Relatively small = 2	105	20.3
	Neutral = 3	185	36.0
	Relatively large = 4	187	36.2
	Very large = 5	15	2.9

**Table 2:** Model variable definitions and assignments.

Categories	Variable Names and Assignment Definitions
Farmers’ characteristics	X1 Gender:
	Male = 1; Female = 0
	X2 Age:
	≤20 = 1; 21~30 = 2; 31~40 = 3; 41~50 = 4; ≥50 = 5
	X3 Education level:
	Primary schools = 1; Junior/middle School =2;
	Technical secondary school and high school = 3; College and above = 4
	X4 Years of agricultural production:
	≤5 = 1; 6~10 = 2; 11~20 = 3; 21~29 = 4; ≥30 = 5
	X5 Planting size:
	≤5 = 1; 6~15 = 2; 16~25 = 3; 25~35 = 4; ≥35 = 5
	X6 Income structure: Agricultural income dominated = 1;
	Non-farm income dominated = 0
	X7 Proportion of pesticide expenditure in total family agricultural expenditure:
	Very small = 1; Relatively small = 2; Neutral =3;
	Relatively large = 4; Very large = 5
Value perception	X8 Perception of degree of income improvement from technology adoption:
	Very small = 1; Relatively small = 2; Neutral = 3;
	Relatively large = 4; Very large = 5
	X9 Perception of technology adoption in improving ecological environment:
	Completely unecological = 1; Relatively unecological = 2;
	Neutral = 3; Relatively ecological = 4; Completely ecological = 5
	X10 Perception of product safety through technology adoption: Very unsafe = 1; Relatively unsafe = 2; Neutral =3; Relatively safe = 4; Completely safe = 5
Risk perception	X11 Risk attitude towards technology adoption:
	Relatively small = 1; Neutral = 2; Relatively large = 3
Ability perception	X12 Perception of easiness in technical operation:
	Very easy = 1; Relatively easy= 2; Neutral = 3; Relatively difficult = 4; Very difficult = 5
Peer influence	X13 Peer influence on your own technology adoption:
	Very small = 1; Relatively small = 2; Neutral = 3;
	Relatively large = 4; Very large = 5
Policy environment	X14 Satisfaction with government subsidies:
	Completely dissatisfied = 1;
	Relatively dissatisfied = 2; Neutral = 3;
	Relatively satisfied = 4; Completely satisfied = 5
	X15 Satisfaction with government technical information publicity:
	Completely dissatisfied = 1; Relatively dissatisfied = 2; Neutral =3;
	Relatively satisfied = 4; Completely satisfied = 5
	X16 Satisfaction with government technical training guidance:
	Completely dissatisfied = 1; Relatively dissatisfied = 2; Neutral =3;
	Relatively satisfied = 4; Completely satisfied = 5
	X17 Trust in government promotion of technology:
	Completely distrust= 1; Relatively distrust = 2; Neutral = 3;
	Relatively trust = 4; Completely trust = 5
	X18 Government’s role in improving the external environment for technology adoption: Very unimportant = 1; Relatively unimportant = 2;
	Neutral = 3; Relatively important = 4; Very important = 5
Relevant experience	X19 Part-time employment:
	Working part-time = 1; Not working part-time = 0
	X20 Membership in professional cooperatives:
	Joined = 1; Did not join = 0
	X21 Frequency of technical problems encountered in industrial operations:
	Encountered = 1; Not encountered = 0
Willingness to adopt	Y1 Willingness to adopt light trapping technology:
	Unwilling = 1; Doesn’t matter = 2; Willing = 3
	Y2 Willingness to adopt biopesticide application technology:
	Unwilling = 1; Doesn’t matter = 2; Willing = 3
	Y3 Willingness to adopt healthy crop growth technology:
	Unwilling = 1; Doesn’t matter = 2; Willing = 3
	Y4 Willingness to adopt insect-proof net technology:
	Unwillingness= 1; Doesn’t matter = 2; Willing = 3

**Table 3:** Farmers’ psychological perceptions of pesticide reduction technologies.

Variables	Classification	Light Trapping Technology		Biological Pesticide Application Technology		Healthy Crop Growth Technology		Insect-Proof Net Technology	
		Frequency	%	Frequency	%	Frequency	%	Frequency	%
Perception of improved agricultural income	Very small = 1;	57	11.0	29	5.6	48	9.3	43	8.3
	Relatively small = 2;	167	32.4	232	45	221	42.8	196	38.0
	Neutral = 3;	241	46.7	199	38.6	126	24.4	228	44.2
	Relatively large = 4;	37	7.2	56	10.9	87	16.9	46	8.9
	Very large = 5	14	2.7	0	0	34	6.6	3	0.6
Perception of improved ecological environment	Completely unecological = 1;	0	0	0	0	0	0	0	0
	Relatively unecological = 2;	18	3.5	58	11.2	2	0.4	15	2.9
	Neutral = 3;	49	9.5	25	4.8	45	8.7	102	19.8
	Relatively ecological = 4;	423	82.0	398	77.1	438	84.9	378	73.3
	Completely ecological = 5	26	5.0	35	6.8	31	6.0	21	4.1
Perception of product safety	Very unsafe = 1;	0	0	0	0	0	0	0	0
	Relatively unsafe = 2;	0	0	6	1.1	0	0	0	0
	Neutral = 3;	17	3.3	17	3.3	28	5.4	17	3.3
	Relatively safe = 4;	478	92.6	472	91.5	465	90.1	479	92.8
	Completely safe = 5	21	4.1	21	4.1	23	4.5	20	3.9
Perception of easiness in technical operation	Very easy = 1;	0	0	0	0	0	0	0	0
	Relatively easy = 2;	175	33.9	61	11.8	181	35.1	130	25.2
	Neutral = 3;	155	30.0	110	21.3	193	37.4	154	29.8
	Relatively difficult = 4;	130	25.2	208	40.3	79	15.3	172	33.3
	Very difficult = 5	56	10.9	137	26.6	63	12.2	60	11.6
Perception of technology adoption risk	Very small = 1;	33	6.4	11	2.1	23	4.5	17	3.3
	Relatively small = 2;	67	13.0	71	13.7	34	6.6	19	3.7
	Neutral = 3;	126	24.4	135	26.2	187	36.2	226	43.8
	Relatively large = 4;	261	50.6	265	51.4	243	47.1	231	44.7
	Very large = 5	29	5.6	34	6.6	29	5.6	23	4.5

**Table 4:** Statistics of farmers’ responsive attitudes to pesticide reduction technologies (number/proportion).

Variable Name	Light Trapping Technology	Biopesticide Application Technology	Healthy Crop Growth Technology	Insect-Proof Net Technology
Heard	338/65.5%	445/87.2%	447/86.6%	487/94.4%
Concerned	167/32.4%	247/47.8%	416/80.6%	378/73.2%
Needed	137/26.6%	118/22.65%	387//74.2%	224/43.4%
Willing to adopt	58/11.2%	81/15.7%	274/53.1%	380/73.6%
Doesn’t matter to adopt	46/8.9%	134/25.9%	104/20.1%	34/6.6%
Unwilling to adopt	412/79.8%	301/58.3%	138/26.7%	102/19.8%

**Table 5:** Correlation analysis of independent variables and farmers’ willingness to respond to light trapping technology.

Independent Variable Name	Maximum Value	Minimal Value	Mean Value	Variance	Kendall’s Tau-b Correlation Coefficient	Significance (Bilateral *p*-Value)
Gender	1.00	0.00	0.548	0.498	−0.113 ***	0.008
Age	5.00	1.00	3.907	1.020	0.124 ***	0.002
Education level	4.00	1.00	1.789	0.824	0.096 **	0.018
Part-time employment	1.00	0.00	0.357	0.479	−0.063	0.114
Professional cooperatives	1.00	0.00	0.614	0.491	0.266 ***	0.000
Years of agricultural production	5.00	1.00	3.859	0.899	0.152	0.231
Planting size	5.00	1.00	2.171	0.933	0.044 **	0.028
Income Structure	2.00	1.00	0.614	0.491	0.073 *	0.090
Proportion of pesticide expenditure	5.00	1.00	2.171	0.934	0.044	0.280
Technology problems	1.00	0.00	0.971	0.028	0.026	0.537
Perception of improved income	5.00	1.00	2.378	0.878	0.632 ***	0.000
Perception of improved environment	5.00	1.00	3.767	0.763	0.012	0.767
Perception of product safety	5.00	1.00	4.014	0.282	0.008	0.841
Peer influence	5.00	1.00	4.174	0.743	0.121 ***	0.004
Operational ability	5.00	1.00	3.364	0.943	−0.047 ***	0.000
Risk perception	3.00	1.00	3.664	2.473	−0.362 ***	0.000
Government subsidies	4.00	1.00	2.324	0.932	0.750 ***	0.000
Information publicity	5.00	1.00	2.244	0.549	0.165 ***	0.000
Technical guidance	5.00	1.00	2.804	0.946	0.750 ***	0.000
Government trust	5.00	1.00	2.248	0.727	0.151 ***	0.000
Government role	5.00	1.00	4.021	0.821	0.109 ***	0.000

Note: ***, **, and * denote significance at the 1%, 5%, and 10% level respectively.

**Table 6:** Estimation result of farmers’ willingness to adopt light trapping technology.

Variable Name	Coefficient	Standard Error	Sig
Perception of improved income	2.086 ***	0.294	0.000
Perception of technology adoption risk	−0.687 ***	0.185	0.000
Government subsidies	2.149 ***	0.324	0.000
Technical guidance	1.642 ***	0.288	0.000
Trust in government promotion of technology	0.696 **	0.303	0.022
Frequency of technical problems encountered	−2.519 **	1.069	0.018
Critical value 1	17.135	3.897	
Critical value 2	8.977	3.761	

Note: Log likelihood = 249.491, LR chi^2^ = 173.567, Prob > chi^2^ = 0.000, Pseudo R^2^ = 0.729. ***, ** denote significance at the 1%, 5% level respectively.

**Table 7:** Estimation result of farmers’ willingness to adopt biopesticide application technology.

Variable Name	Coefficient	Standard Error	Sig
Perception of improved income	2.197 ***	0.838	0.000
Perception of technology adoption risk	−1.644 **	0.719	0.022
Government subsidies	4.019 ***	0.957	0.000
Technical guidance	5.716 ***	1.189	0.000
Trust in government promotion of technology	1.997 ***	0.740	0.007
Perception of government in improving the environment for technology adoption	4.533 ***	1.111	0.000
Critical value 1	46.394	13.882	
Critical value 2	27.936	12.415	

Note: Log likelihood = 508.719, LR chi^2^ = 152.694, Prob > chi^2^ = 0.000, Pseudo R^2^ = 0.256. ***, ** denote significance at the 1%, 5% level respectively.

**Table 8:** Estimation result of farmers’ willingness to adopt healthy crop growth technology.

Variable Name	Coefficient	Standard Error	Sig
Perception of improved income	8.414 ***	1.466	0.000
Perception of technical operational ability	−2.455 ***	0.686	0.000
Government subsidies	2.509 ***	0.758	0.001
Technical guidance	5.496 ***	1.127	0.000
Trust in government promotion oftechnology	1.850 **	0.786	0.019
Critical value 1	7.773	1.394	
Critical value 2	5.596	1.192	

Note: Log likelihood = 624.094, LR chi^2^ = 414.969, Prob > chi^2^ = 0.000, Pseudo R^2^ = 0.553. ***, ** denote significance at the 1%, 5% level respectively.

**Table 9:** Estimation result of farmers’ willingness to adopt insect-proof net technology.

Variable Name	Coefficient	Standard Error	Sig
Age	1.001 ***	0.288	0.001
Years of agricultural production	0.622 ***	0.238	0.009
Perception of improved income	3.737 ***	0.406	0.000
Perception of technical operational ability	−0.741 ***	0.167	0.000
Perception of technology adoption risk	−2.294 ***	0.230	0.000
Government subsidies	1.752 ***	0.312	0.000
Trust in government promotion of technology	0.986 ***	0.223	0.000
Critical value 1	3.602	3.289	
Critical value 2	9.115	3.331	

Note: Log likelihood = 624.094, LR chi^2^ = 414.969, Prob > chi^2^ = 0.000, Pseudo R^2^ = 0.553. *** denote significance at the 1% level respectively.

## Data Availability

The data presented in this study are mainly from a field survey of farmers conducted by the research team in Kaifeng, Henan Province from July to September 2020, which was conducted by questionnaires and interviews by the researchers after the training. Based on the principle of random sampling, six villages in the administrative area of Kaifeng were randomly selected for field research in this study. A total of 516 valid questionnaires were retained for this survey.

## Supplementary Materials

The following supporting information can be downloaded at: https://www.mdpi.com/article/10.3390/ijerph19095136/s1. Farmers’ Green Farming Technology Adoption Questionnaire.

## References

[1] Gao Y., Zhang X., Lu J., Wu L., Yin S. (2017). Adoption behavior of green control techniques by family farms in China: Evidence from 676 family farms in Huang-huai-hai Plain. Crop Prot..

[2] Tessema R.A., Nagy K., Ádám B. (2021). Pesticide Use, Perceived Health Risks and Management in Ethiopia and in Hungary: A Comparative Analysis. Int. J. Environ. Res. Public Health.

[3] Han H., Cai S. (2011). Research on the health cost of pesticide application and its influencing factors—Based on the research data of farmers in the main grain producing areas. J. Chin. Agric. Univ..

[4] Cai R., Wang Z., Qian L. (2019). Does joining cooperatives promote family farms to choose environmentally friendly production methods? —Taking fertilizer and pesticide application reduction as an example. Chin. Rural Surv..

[5] Xue B., Zheng S. (2019). A study on farmers’ production technology selection behavior under the threshold of agricultural product quality and safety: An example of kiwifruit farmers in Shaanxi Province. J. Northwest A F Univ..

[6] Tian Y., Zhang J., He K. (2015). Analysis of farm households’ agricultural low-carbon production behavior and its influencing factors —Taking fertilizer application and pesticide use as examples. Chin. Rural Surv..

[7] Xia Q., Li D., Zhou H. (2018). Study on the impact of farming part-time on agricultural surface source pollution. Chin. Popul., Resour. Environ..

[8] Li R., Chen Y. (2017). Research on farmland conservation and utilization behavior of farming households in typical black soil areas in Northeast China—An empirical analysis based on a survey of farming households in Suihua City, Heilongjiang Province. J. Agrotech. Econ..

[9] Cai R., Cai S. (2012). An empirical analysis of conservation tillage technology adoption and its impact on crop yields—Based on survey data from rice farmers in Anhui Province. Resour. Sci..

[10] Zhao P., Yan B., Liu T. (2021). Socio-economic status differences and the inverted U-shaped relationship between green control technology diffusion in agriculture: The mediating effect of social learning. J. Arid Land Resour. Environ..

[11] Huang Y., Luo X., Tang L. (2020). The cost-saving and income-generating effects of green control technologies. Chin. Popul. Resour. Environ..

[12] Guo L., Wang Y. (2018). Why do farmers say one thing but do another in the application of biopesticides?. J. Huazhong Agric. Univ..

[13] Wang X., Zhang J., He K., He P. (2020). The influence of risk perception and public image appeal on farmers’ adoption of green farming technologies. J. Chin. Agric. Univ..

[14] Zhao Q., Xia X. (2020). How social norms influence farmers’ pesticide application reduction—An analysis of the moderating effect based on the mediating effect of moral responsibility and socio-economic status differences. J. Agrotech. Econ..

[15] Wang H., Xu X. (2004). Micro-behavior and agricultural product safety: An analysis of farmers’ production and consumers’ consumption. J. Nanjing Agric. Univ..

[16] Galt R.E. (2007). Regulatory risk and farmers’ caution with pesticides in Costa Rica. Trans. Inst. Br. Geogr..

[17] Pimentel D., Acquay H., Biltonen M., Rice P., Silva M., Nelson J., Lipner V., Giordano S., Horowitz A., D’Amore M. (1992). Environmental and economic costs of pesticide use. Bioscience.

[18] Eskenazi B., Bradman A., Castorina R. (1999). Exposures of children to organophosphate pesticides and their potential adverse health effects. Environ. Health Perspect..

[19] Ma W. (2022). Epidemiological analysis of pesticide poisoning in Chongming District, Shanghai, 2007–2019. Occup. Health.

[20] Liu S., Shen X., Zhu S. (2020). Research on the green production behavior of farmers under the evolution of agricultural industrialized management organization system. Rural Econ..

[21] Cheng L., Zhang J., He K. (2019). Analysis of the influence of network embedding and risk perception on farmers’ green farming technology adoption behavior—Based on survey data from 615 farmers in Hubei Province. Resour. Environ. Yangtze Basin.

[22] Li C., Zhou H. (2021). Organizational embedding and pesticide reduction among farmers: An analysis based on rice farmers in Jiangsu Province. Res. Agric. Mod..

[23] Zhang L., Luo B. (2019). Agricultural reduction and its path options: Evidence from green energy companies. Rural Econ..

[24] Gai H., Yan T., He K., Zhang J. (2019). Research on farmers’ conservation tillage technology adoption behavior from the perspective of social embeddedness—Based on data from 668 farmer surveys in three provinces of Ji, Wan and E. Resour. Environ. Yangtze Basin.

[25] Huang Y., Luo X., Li R., Zhang J. (2018). Farmers’ perceptions, external environment and willingness to produce green agriculture: Based on research data from 632 farmers in Hubei Province. Resour. Environ. Yangtze Basin.

[26] Li F., Zhang J., He K. (2019). Substitution and complementarity: Informal and formal institutions in farmers’ green production. J. Huazhong Univ. Sci. Technol..

[27] Xiang C., Ji N. (2021). The influence of social capital on farmers’ willingness to adopt fertilizer and pesticide reduction technologies-mediated by learning ability and moderated by ecological cognition. J. Chin. Agric. Univ..

[28] Li X., Zhang L. (2019). An empirical analysis of farmers’ willingness to adopt cleaner production technologies and the influencing factors. Res. Agric. Mod..

[29] Wu B. (2014). A review of green consumption research. Bus. Manag. J..

[30] Yang G., Zhang L., Yue M., Zhang J. (2020). Can socialized agricultural services promote reduced agricultural production-an empirical analysis based on micro-survey data of rice farmers in the Jianghan Plain. World Agric..

[31] Wu Y., Xi X., Tang X., Luo D., Gu B., Lam S.K., Chen D. (2018). Policy distortions, farm size, and the overuse of agricultural chemicals in China. Proc. Natl. Acad. Sci. USA.

[32] Guo Q., Li S., Nan L. (2020). Social learning, social networks and pesticide reduction-empirical evidence from micro data of farmers. J. Arid Land Resour. Environ..

[33] Zhang L. (2020). Smallholder differentiation, behavioral differences, and agricultural reduction. Iss. Agric. Econ..

[34] Yang Y., He Y., Yan G. (2021). The impact of different incentives on farmers’ green production behaviors: An example of biopesticide application. World Agric..

[35] Li F., Zhang J., He K. (2019). Influence of informal institutions and environmental regulations on farmers’ green production behavior: Based on 1105 farmers’ survey data in Hubei. Resour. Sci..

[36] Qin S., Lv X. (2020). Can the participation of market players reduce pesticide overuse by rice farmers?. J. Huazhong Agric. Univ..

[37] Wei W. (2021). Practice and prospect of pesticide use reduction in China. Agric. Technol..

[38] Yan A., Luo S., Huang Y. (2021). Influence of socialized services on farmers’ pesticide reduction behavior. J. Arid Land Resour. Environ..

[39] Porter M.E. (1985). Competitive Advantage.

[40] Zeithaml V.A. (1988). Consumer Perceptions of Price, Quality and Value: A Means-End Model and Synthesis of Evidence. J. Mark..

[41] Monroe G.C., Benbasat I. (1991). Development of An Instrument to Measure the Perceptions of Adopting an Information Technology Innovation. Inf. Syst. Res..

[42] Gronroos C. (1997). Value-Driven Relational Marketing: From Products to Resources and Competencies. J. Mark. Manag..

[43] Petrick J.F. (2002). Development of A Multi-Dimensional Scale for Measuring the Perceived Value of a Service. J. Leisure Res..

[44] Wu X., Zhang J., Feng J. (2017). Factors influencing farmers’ green farming technology perception and its hierarchical structure decomposition—Based on Probit-ISM model. J. Huazhong Agric. Univ..

[45] Shen M., Gan C., Chen Y., Mei Y. (2019). Research on factors influencing farmers’ willingness to transfer farmland out based on DTPB theory—An example from Wuhan city circle. Res. Agric. Mod..

[46] Ren L., Lin Z., Li N. (2018). A study on residents’ willingness to pay for ecological watershed governance. Res. Financ. Educ..

[47] Wu J., Zan M., Wang Z. (2020). Influence of perceived value on farmers’ willingness to participate in arable land quality conservation: The case of Shaanxi Province. Chin. Land Sci..

[48] Biswas A., Roy M. (2015). Leveraging factors for Sustained green consumption behavior based on consumption value perceptions: Testing the structural model. J. Clean. Prod..

[49] Zhang J., Yan T., Jiang X. (2021). Value perceptions, environmental responsibility awareness and straw resource utilization by farmers—A multi-group analysis based on an extended technology acceptance model. Chin. J. Agric. Resour. Reg. Plan..

[50] Zhao H., Hu W. (2021). The influence of farmers’ perceptions on their willingness to participate in agricultural waste resource utilization under environmental regulations. Chin. J. Eco-Agric..

[51] Cao H., Zhao K. (2018). Factors influencing farmers’ intention to reduce fertilizer application and decomposition of its effect—An empirical analysis based on VBN-TPB. J. Huazhong Agric. Univ..

[52] Yang F., Zheng X. (2021). The influence of ecological compensation methods on farmers’ green production behavior from the perspective of value perception. Chin. Popul., Resour. Environ..

[53] Mo J., Yu Z. (2020). Influence of value perception on farmers’ propensity to adopt technology and its conditional response—Empirical evidence based on structural equation modeling. Chin. J. Agric. Resour. Reg. Plan..

[54] Ajzen I. (1991). The Theory of Planned Behavior. Organ. Behav. Hum. Dec. Processes.

[55] Hagger M., Chatzisarantis N. (2005). First -and higher-order models of attitudes, normative influence, and perceived behavioral control in the theory of planned behavior. Br. Psychol. Soc..

[56] Greaves M., Zibarras L.D., Stride C. (2013). Using the theory of planned behavior to explore environmental behavioral intentions in the workplace. J. Environ. Psychol..

[57] Xie X., Chen M. (2019). Analysis of farmers’ willingness to adopt ecological farming and its heterogeneity—An empirical study based on TPB framework. Resour. Environ. Yangtze Basin.

[58] Hu N., Wang Y., Chen Q., Zhu L. (2021). Factors influencing farmers’ willingness to adopt rice and shrimp crop models and their heterogeneity. Chin. J Eco-Agric..

[59] Ruan W., Yu H., Song X. (2019). A study on the behavioral intention of international tourists under the threat of haze based on the theory of extended planned behavior—An example of international tourists in Beijing. J. Arid Land Resour. Environ..

[60] Shi Z., Cui M., Zhang H. (2020). A study on farmers’ willingness to produce green based on extended theory of planned behavior. J. Arid Land Resour. Environ..

[61] Shi P., Yu J. (2021). The influence of risk expectation, citizenship perception and farmers’ cognition on the withdrawal of homestead bases of farmers relocated to alleviate poverty. Resour. Sci..

[62] Guo Q., Li H., Li S. (2019). Influence of social norms on adoption behavior of fertilizer reduction measures by farm households. J. Northwest A F Univ..

[63] Gao J., Shi Q. (2019). The influence of farmers’ productive characteristics on pesticide application: Mechanisms and evidence. Chin. Rural Econ..

[64] Li F., He K., Zhang J. (2021). The influence of value perception on the willingness of large-scale pig farmers to participate in agricultural carbon trading and the expected carbon price: An example of a household biogas CCER project. Res. Agric. Mod..

[65] Zhang T., Yan T., He K., Zhang J. (2019). Contrary of farmers’willingness of straw utilization to the behavior: Based on the MOA model. J. Arid Land Resour. Environ..

[66] Su S., Zhou X., Zhou Y. (2022). Can organic fertilizer substitution increase farmers’ income?—A case study of vegetable growers in Shandong. J. Arid Land Resour. Environ..

[67] He L., Wang Y. (2019). Subsidies on organic fertilizer, perception of the effect of organic fertilizer use and farmers’use of organic fertilizer—A survey based on some pilot and non-pilot counties of organic fertilizer subsidies in Shaanxi Province. J. Arid Land Resour. Environ..

[68] He P., Zhang J., He K., Zeng Y. (2019). Cognitive social capital and farmers’ participation behavior in environmental governance—An example of straw resource utilization approach. Chin. J. Agric. Resour. Reg. Plan..

[69] Huang Y., Luo X. (2018). Eating and selling at the same time: An analysis of differences in biopesticide application behavior of rice farmers. Chin. Rural Econ..

[70] Guo L., Zhao J. (2017). Research on farmers’ willingness to apply biopesticides from the perspective of cognitive conflict—Empirical evidence based on 639 rice farmers in Jiangsu. J. Nanjing Agric. Univ..

[71] Wang J., Wang Q., Zhao Q. (2014). Current status and countermeasures for the development of professional pest control in China. Chin. Plant Protect..

[72] Meng Y. (2021). The promotion and application of biopesticide technology in agricultural cultivation in the new era. Seed Sci. Technol..

[73] Du S., Luo X., Huang Y., Tang L., Yu W. (2021). Risk perception, agricultural social services and rice farmers’ biopesticide technology adoption behavior. Resour. Environ. Yangtze Basin.

[74] Liu Y. (2012). Application status and development trend of light pest control technology. J. Chin. Agric. Mech..

[75] Sang W., Huang Q., Wang X., Guo S., Lei C. (2019). Development, achievements and prospects of phototropism of insects and light trapping technology in China. Chin. J. Appl. Entomol..

[76] Li W., Zhang W., Wang L., Jin Y. (2018). Analysis of the main influencing factors of pesticide use in Shanxi Province and discussion of countermeasures to reduce the amount. Chin. Plant Protect..

[77] Guo Z., Xie Y., Xu R., Wang J., Xie H., Zhang Z. (2020). Practice and reflection on the promotion of pesticide reduction in Hubei Province. Chin. Plant Protect..

[78] Yang P., Wang K., Li J., Li W., Yin J. (2018). Helping agricultural green development with pesticide reduction and pest control. Plant Protect..

[79] Wang X., Li Z., Tan X., Lei Q., Shao C., Jiao M., Wu Q. (2021). Measures and effectiveness of zero growth action of pesticide use in Guizhou Province. Chin. Plant Protect..

[80] Jin X., Zhang Q., Yao Z., Zhu W. (2020). Survey and analysis of pesticide use in Tongxiang City. Chin. Plant Protect..

[81] Xiong Y., Li X., Zhong Y. (2021). A study on farmers’ application behavior based on reduction targets—Micro data from grain farmers in seven provinces. Chin. J. Eco-Agric..

