# Supplementary material for: More Income, Less Pollution? How Income Expectation Affects Pesticide Application
Source: Int J Environ Res Public Health. 2022 Apr 23;19(9):5136. doi: 10.3390/ijerph19095136 (PMC9099653; doi:10.3390/ijerph19095136)
Supplement: Supplementary file 1 [file ijerph-19-05136-s001.zip › ijerph-1640117-supplementary.pdf]

Supplementary Materials:

Farmers' Green Farming Technology Adoption Questionnaire

Sincerely thank you for filling in the questionnaire during your busy schedule!!! (This questionnaire is for academic research)

Name of responden: ____________

____________ Province ____________ County (City) ____________ Town ______

Village ________ Group ___

(Please put your actual situation and ideas directly on the number or symbol with "√", and fill in the corresponding information in the blank. Thank you!)

I. Basic information about the farmer's household

Your total household size is____, of which: household labor force is____ and outworking labor force is____.

**Family Member Code**	**1**	**2**	**3**
**Relationship with the head of household**<br>A = Household head; B = Couple; C = Father and son; D = Mother and son; E = Father and daughter; F = Mother and daughter; G = Other	Household head (A)		
**Gender**<br>A = Male; B = Female			
**Age**<br>A = 20 and below; B = 21–30; C = 31–40; D = 41–50; E = 51–65; F = 66 and above			
**Education level**<br>A = No or little literacy; B = Primary school; C = Junior/middle school; D = Technical secondary school and high school; E = College and above			
**Years of agricultural production**<br>A = 10 years and below; B = 11–20; C = 21–30; D = 31–40; E = 41–50; F = 51 years and above			
**Other part-time employment**<br>A = Village officials; B = Technician; C = Village teacher; D = Enterprise worker; E = Village doctor; F = Other part-time work; G = No part-time work			
**Part-time employment income (ten thousand yuan/year)**<br>A = 0.5 and below; B = 0.5–1; C = 1–2; D = 2–3; E = 3–4; F = 4 and above			
**Part-time employment time (months)**<br>A = 1 and below; B = 1–2; C = 2–3; D = 3–4; E = 4–5; F = 5 and above			

Note: If the respondent is the household head, please fill in column "1"; if the respondent is not the household head, please fill in column "2" with the information about yourself and column "1" with the basic information about the household head.

II. Scale of family business and income and expenditure (2020)

1. In 2019, your family' s contracted land has a total of ____ mu.

Among them: ____ mu of paddy field, totaling ____ blocks (hills); ____ mu of dry land, totaling ____ blocks (hills).

2. How is the contracted land handled now?

A. Own business ____ mu

B. Abandoned

C. Free transfer to others to plant ____ mu

D. Leased to others for a fee ____ mu ____ years, the cost (____ yuan/mu·year)

E. Other

3. Your family's annual income is ____ ten thousand yuan, of which agriculture income is ____ ten thousand yuan.

4. Your family's annual expenditure is ____ ten thousand yuan, of which agriculture expenditure is ____ ten thousand yuan. The largest agricultural expenses are mainly for ( )

A. Fertilizer
B. Pesticides
C. Seed purchase
D. Agricultural machinery for cultivation and harvesting
E. Other

5. The main type of agricultural business of your family is ( )

A. Crops
B. Fruits and vegetables
C. Livestock and poultry
D. Fishery
E. Agricultural products processing

6. Have you joined local farmers' professional cooperatives?

A. Yes
B. No (If not, please ignore questions 7 and 8)

7. Why do you join farmers' professional cooperatives? ( )

A. Voluntary participation, think it is beneficial
B. Follow the crowd
C. Village cadres request

8. Do you think joining farmers' professional cooperatives helps you a lot in your production and operation? ( )

A. Very helpful
B. Relatively helpful
C. Neutral
D. Relatively not helpful
E. No helpful at all

9. If helpful, in what ways? Multiple choices are allowed ( )

A. Access to new technology information
B. Technical guidance and demonstration
C. Centralized procurement of pesticides and fertilizers
D. Arrangement of mechanized farming
E. Centralized application of pesticides and fertilizers
F. Active help in finding sales channels for agricultural products

III. Perception and adoption of pesticide-saving green agricultural technologies (2020)

1. Please tick the options of the pesticide-saving green agricultural technologies you have heard of. Multiple choices are allowed. ( )

A. According to the occurrence law of crop diseases and insect pests, choose the right time, the right amount and the right variety of pesticide application technology
B. The remote monitoring and early-warning technology for plant diseases and insect pests
C. Seed coating technology
D. Specialized integrated control of plant protection
E. Using substitutional and high activity pesticide (e.g., alika (thiamethoxam and lambda cyhalothrin), xiaochongthion, sulfoxime, furan tebufenozide, chloramine phosphorus, butylene fipronil, etc.)
F. Using growth regulators and pesticide synergists (e.g., available diethyl aminoethyl hexanoate (DA-6), forchlorfenuron, compound sodium nitrophenolate, brassins, gibberellic acid, etc.)
G. Using the technology of healthy crop growth (e.g., soil test and formula fertilization, crop rotation and intercropping, deep loosening and tilling of the soil, etc.)

H. Using efficient plant equipment to precisely apply pesticides
I. Biopesticide (e.g., bacillus thuringiensis, bacillus subtilis, bacillus cereus, helicoverpa armigera nucleopolyhedrovirus (HearNPV); oligosaccharins, chitosan, eugenol, 5-Aminolevulinic Acid hydrochloride, boscalid; nicotine, rotenone, matrine, pyrethroid, azadirachtin, brassinolide, ethylicin; avermectin, jinggangmycin, kasugamycin, etc.)
J. Light trap
K. Sticky trap
L. Insect sex pheromone trap
M. Natural enemy insect
N. Inducers
O. Insect-proof net
2. How well do you know about the above-mentioned pesticide-saving green agricultural technologies that you have heard of? ( )
A. Completely unfamiliar
B. Relatively unfamiliar
C. Neutral
D. Relatively familiar
E. Completely familiar
3. Do you need such technologies? ( )
A. Yes
B. No
C. Not sure
4. Why do you need them? ( )
A. Improve yield and quality
B. Reduce toxicity and residue
C. Protect the environment
D. Increase income
E. Safe for health
F. Others are using it
5. Why do you not need them? ( )
A. The price is too high
B. The effect is not significant
C. Do not know how to operate
D. No one else uses it
E. The environment factors are not very relevant to me
6. Through which channels did you learn about these technologies? ( ); Through what channels do you think it is best to learn about these technologies? ( )
A. Lectures by relevant government departments
B. Technical extension and training of agricultural technology extension agencies
C. TV news, radio, WeChat and other media
D. Technical manuals, books, etc.
E. Oral recommendation from family members, friends and neighbors
F. Seeing farmers using it with your own eyes
G. Searching online for relevant technical information
7. Do you use these technologies? ( )
A. Yes
B. No
8. If your choice is yes, what technologies have you used? ( ); Which technologies are you going to try next? ( ); Which technologies are you not willing to use? ( ) Multiple choices are allowed.
A. According to the occurrence law of crop diseases and insect pests, choose the right time, the right amount and the right variety of pesticide application technology
B. The remote monitoring and early-warning technology for plant diseases and insect pests

C. Seed coating technology
D. Specialized integrated control of plant protection
E. Using substitutional and high activity pesticide (e.g., alika (thiamethoxam and lambda cyhalothrin), xiaochongthion, sulfoxime, furan tebufenozide, chloramine phosphorus, butylene fipronil, etc.)
F. Using growth regulators and pesticide synergists (e.g., available diethyl aminoethyl hexanoate (DA-6), forchlorfenuron, compound sodium nitrophenolate, brassins, gibberellic acid, etc.)
G. Using the technology of healthy crop growth (e.g., soil test and formula fertilization, crop rotation and intercropping, deep loosening and tilling of the soil, etc.)
H. Using efficient plant equipment to precisely apply pesticides
I. Biopesticide (e.g., bacillus thuringiensis, bacillus subtilis, bacillus cereus, helicoverpa armigera nucleopolyhedrovirus (HearNPV); oligosaccharins, chitosan, eugenol, 5-Aminolevulinic Acid hydrochloride, boscalid; nicotine, rotenone, matrine, pyrethroid, azadirachtin, brassinolide, ethylicin; avermectin, jinggangmycin, kasugamycin, etc.)
J. Light trap
K. Sticky trap
L. Insect sex pheromone trap
M. Natural enemy insect
N. Inducers
O. Insect-proof net
9. Do you think these green agricultural technologies are easy to operate? ( )
A. Very easy
B. Relatively easy
C. Neutral
D. Relatively difficult
E. Very difficult
10. Have you encountered any technical problems in agricultural production? ( )
A. Yes
B. No
11. How many times have you attended training on pesticide-saving green agricultural technologies by agricultural technology extension agencies? ( )
A. Never
B. 1–3 times
C. 4–6 times
D. 7–10 times
E. 10–15 times
F. More than 15 times
12. Are you satisfied with the training on pesticide-saving green agricultural technologies provided by agricultural technology extension agencies? ( )
A. Very satisfied
B. Relatively satisfied
C. Neutral
D. Relatively dissatisfied
E. Very dissatisfied
13. What improvements would you like to see in these technical trainings? Multiple choices are allowed ( )
A. More field demonstrations
B. More detailed explanations
C. More frequent training
D. Better trainers
E. More flexible training time, preferably short-term training during the agricultural leisure periods
14. In what way would you like the technical training to be conducted? ( )

A. Online teaching video
B. Real-time WeChat connection for remote expert consultation
C. Offline technical training courses
D. Technical guidance in the field
E. On-site observation and learning at the homes of demonstration households
F. One-to-one guiding from technicians in the countryside
15. Do you think the impact of adopting pesticide-saving green agricultural technologies on income improvement is large? ( )
A. Very small
B. Relatively small
C. Neutral
D. Relatively large
E. Very large
16. Do you think the impact of adopting pesticide-saving green agricultural technologies on improving the ecological environment is ecological enough? ( )
A. Completely unecological
B. Relatively unecological
C. Neutral
D. Relatively ecological
E. Completely ecological
17. Do you think the impact of adopting pesticide-saving green agricultural technologies on product safety is safe? ( )
A. Very unsafe
B. Relatively unsafe
C. Neutral
D. Relatively safe
E. Very safe
18. Do you think the risk of adopting pesticide-saving green agricultural technologies is large? ( )
A. Relatively small
B. Neutral
C. Relatively large
19. How large is the impact of the people around you adopting technology on you own decision? ( )
A. Very small
B. Relatively small
C. Neutral
D. Relatively large
E. Very large
20. Are you satisfied with the government's technology adoption subsidies? ( )
A. Very satisfied
B. Satisfied
C. Neutral
D. Dissatisfied
E. Very dissatisfied
21. Do you trust the pesticide-saving green agricultural technologies promoted by relevant government departments? ( )
A. Completely distrust
B. Relatively distrust
C. Neutral
D. Relatively trust
E. Completely trust

22. How improtant do you think the government' role in improving the external environment for technology adoption? ( )
A. Very unimportant
B. Relatively unimportant
C. Neutral
D. Relatively important
E. Very important
IV. Perception and adoption of fertilizer-saving green agricultural technologies (2020)
1. Please tick the option of fertilizer reduction and efficiency technologies you have heard of. Multiple choices are allowed.
A. According to the law of crop growth, choose the right time, the right amount and the right variety of pesticide application technology
B. Soil test and formula fertilization technology
C. The technology of slow and controlled release fertilizer
D. Efficient mechanized fertilization technology (e.g., mechanized seed and fertilizer co-sowing, mechanized deep fertilization technology, layered fertilization and sowing of subsoiling, integrated irrigation with water and fertilizer, etc.)
E. The technology of straw return to soil
F. The technology of biogas residue and biogas slurry returning to soil
G. The technology of healthy crop growth (e.g., crop rotation and intercropping, deep loosening and tilling of the soil, etc.)
2. How well do you know about the above-mentioned fertilizer-saving green agricultural technologies that you have heard of? ( )
A. Completely unfamiliar
B. Relatively unfamiliar
C. Neutral
D. Relatively familiar
E. Completely familiar
3. Do you need such technology? ( )
A. Yes
B. No
C. Not sure
4. Why do you need them? ( )
A. Improve yield and quality
B. Reduce toxicity and residue
C. Protect the environment
D. Increase income
E. Safe for health
F. Others are using it
5. Why do you not need them? ( )
A. The price is too high
B. The effect is not significant
C. Do not know how to operate
D. No one else uses it
E. The environment factors are not very relevant to me
6. Through which channels did you learn about these technologies? ( ); Through what channels do you think it is best to learn about these technologies? ( )
A. Lectures by relevant government departments
B. Technical extension and training of agricultural technology extension agencies
C. TV news, radio, WeChat and other media
D. Technical manuals, books, etc.
E. Oral recommendation from family members, friends and neighbors

F. Seeing farmers using it with your own eyes
G. Searching online for relevant technical information
7. Do you use these technologies? ( )
A. Yes
B. No
8. If your choice is yes, what technologies have you used? ( ); Which technologies are you going to try next? ( ); Which technologies are you not willing to use? ( ) Multiple choices are allowed.
A. According to the law of crop growth, choose the right time, the right amount and the right variety of pesticide application technology
B. Soil test and formula fertilization technology
C. The technology of slow and controlled release fertilizer
D. Efficient mechanized fertilization technology (e.g., mechanized seed and fertilizer co-sowing, mechanized deep fertilization technology, layered fertilization and sowing of subsoiling, integrated irrigation with water and fertilizer, etc.)
E. The technology of straw return to soil
F. The technology of biogas residue and biogas slurry returning to soil
G. The technology of healthy crop growth (e.g., crop rotation and intercropping, deep loosening and tilling of the soil, etc.)
9. Do you think these green agricultural technologies are easy to operate? ( )
A. Very easy
B. Relatively easy
C. Neutral
D. Relatively difficult
E. Very difficult
10. Have you encountered any technical problems in agricultural production? ( )
A. Yes
B. No
11. How many times have you attended training on pesticide-saving green agricultural technologies by agricultural technology extension agencies? ( )
A. Never
B. 1–3 times
C. 4–6 times
D. 7–10 times
E. 10–15 times
F. More than 15 times
12. Are you satisfied with the training on fertilizer-saving green agricultural technologies provided by agricultural technology extension agencies? ( )
A. Very satisfied
B. Relatively satisfied
C. Neutral
D. Relatively dissatisfied
E. Very dissatisfied
13. What improvements would you like to see in these technical trainings? Multiple choices are allowed ( )
A. More field demonstrations
B. More detailed explanations
C. More frequent training
D. Better trainers
14. Do you think the impact of adopting fertilizer-saving green agricultural technologies on income improvement is large? ( )
A. Very small
B. Relatively small

C. Neutral
D. Relatively large
E. Very large

15. Do you think the impact of adopting fertilizer-saving green agricultural technologies on improving the ecological environment is ecological enough? ( )

A. Completely unecological
B. Relatively unecological
C. Neutral
D. Relatively ecological
E. Completely ecological

16. Do you think the impact of adopting fertilizer-saving green agricultural technologies on product safety is safe? ( )

A. Very unsafe
B. Relatively unsafe
C. Neutral
D. Relatively safe
E. Very safe

17. Do you think the risk of adopting fertilizer-saving green agricultural technologies is large? ( )

A. Relatively small
B. Neutral
C. Relatively large

18. How large is the impact of the people around you adopting technology on your own decision? ( )

A. Very small
B. Relatively small
C. Neutral
D. Relatively large
E. Very large

19. Do you trust the fertilizer-saving green agricultural technologies promoted by relevant government departments? ( )

A. Completely distrust
B. Relatively distrust
C. Neutral
D. Relatively trust
E. Completely trust

20. How important do you think the government' important role in improving the external environment for technology adoption? ( )

A. Very unimportant
B. Relatively unimportant
C. Neutral
D. Relatively important
E. Very important

21. What are the government policies that you know about adopting fertilizer and pesticide-saving technologies for agricultural benefit? (e.g., subsidies for the purchase of agricultural machinery for green agriculture; subsidies for organic fertilizer to replace chemical fertilizer technology, physical and chemical subsidies, etc.) Are you satisfied? ( ) If not, why?

A. Very satisfied
B. Satisfied
C. Neutral
D. Dissatisfied
E. Very dissatisfied
